# Risk and promise: an 11-year, single-center retrospective study of severe acute GVHD in pediatric patients undergoing allogeneic HSCT for nonmalignant diseases

**DOI:** 10.3389/fped.2023.1194891

**Published:** 2023-05-26

**Authors:** Irina Zaidman, Ehud Even-Or, Elroee Aharoni, Dina Averbuch, Yael Dinur-Schejter, Adeeb NaserEddin, Mordechai Slae, Bella Shadur, Polina Stepensky

**Affiliations:** ^1^ Faculty of Medicine, Hebrew University of Jerusalem, Jerusalem, Israel; ^2^ Department of Bone Marrow Transplantation and Cancer Immunotherapy, Hadassah-Hebrew University Medical Center, Jerusalem, Israel; ^3^ Department of Pediatrics, Hadassah-Hebrew University Medical Center, Jerusalem, Israel; ^4^ Immunology Division, Garvan Institute of Medical Research, Sydney, NSW, Australia; ^5^ St Vincent’s Clinical School, University of New South Wales, Sydney, NSW, Australia

**Keywords:** hematopoietic stem cell transplantation, survival, graft-vs.-host disease, pediatric nonmalignant diseases, outcome

## Abstract

**Background:**

Hematopoietic stem cell transplantation (HSCT) is the only curative option for many nonmalignant hematopoietic-derived diseases in pediatric patients. Survival after HSCT has improved in recent years and resulted in a 90% survival rate and cure in some nonmalignant diseases. Graft-vs.-host disease (GVHD) remains a frequent and major complication of HSCT, and a leading cause of morbidity and mortality. Prognosis of patients with high-grade GVHD is dismal, with survival rates varying from 25% in the adult population to 55% in pediatric patients.

**Methods:**

The main aim of this study is to evaluate the incidence, risk factors, and outcome of severe acute GVHD (AGVHD) in pediatric patients with nonmalignant diseases, following allogeneic HSCT. Clinical and transplant data were retrospectively collected for all pediatric patients who underwent allogeneic HSCT for nonmalignant diseases at the Hadassah Medical Center between 2008 and 2019. Patients who developed severe AGVHD were compared with those who did not.

**Results:**

A total of 247 children with nonmalignant diseases underwent 266 allogeneic HSCTs at Hadassah University Hospital over an 11-year period. Seventy-two patients (29.1%) developed AGVHD, 35 of them (14.1%) severe AGVHD (grade 3–4). Significant risk factors for developing severe AGVHD were unrelated donor (*p* < 0.001), mismatch donor (*p* < 0.001), and the use of peripheral blood stem cells (PBSCs) (*p* < 0.001). Survival rates of pediatric patients with severe AGVHD was 71.4%, compared with 91.9% among those with mild (grade 1–2) AGVHD and 83.4% among patients without AGVHD (*p* = 0.067).

**Conclusions:**

These results demonstrate a high survival rate in pediatric patients with nonmalignant diseases despite severe GVHD. Significant mortality risk factors found in these patients were the source of donor PBSC (*p* = 0.016) and poor response to steroid treatment (*p* = 0.007).

## 1. Introduction

Hematopoietic stem cell transplantation (HSCT) remains the only curative option for many malignant and nonmalignant diseases in pediatric patients. Graft-vs.-host disease (GVHD) remains a major complication of HSCT and a leading cause of morbidity and mortality. Prognosis of patients with high-grade GVHD is dismal and survival rates vary between 25% in adult populations and 55% in pediatric patients (1).

GVHD is an immunologically mediated process involving donor immune cell responses against major or minor histocompatibility antigens of the recipient. Acute GVHD (AGVHD) is driven by donor lymphocytes and the inflammatory cytokine cascade (2, 3). In children with malignant diseases, graft-vs.-host effect may correlate with graft-vs.-leukemia effect and reduce the risk of relapse. However, there is no beneficial role for GVHD in patients with nonmalignant disorders (3).

Among all patients undergoing allogeneic HSCT, 30%–50% develop AGVHD and 14% develop severe AGVHD (grade 3–4) (4). Risk factors include the degree of human leukocyte antigen (HLA) mismatch, receipt of a transplant from an unrelated donor (UD), a female donor to a male recipient, the use of peripheral blood stem cell (PBSC) grafts, and the intensity of the conditioning regimen (5). Higher grades of AGVHD have consistently been associated with worse transplant-related mortality (TRM) and lower overall survival (OS) rates (6).

AGVHD principally involves the skin, gastrointestinal tract (GIT) and liver, with skin manifestations occurring more commonly and usually the earliest following engraftment (7–9). Patients typically develop a pruritic maculopapular rash, initially around the neck and shoulders, often involving the palms and soles but sparing the scalp. In severe cases, blistering and ulceration can occur. GIT GVHD usually presents with diarrhea, but may also manifest as vomiting, nausea, anorexia, abdominal pain, and, in severe cases, GIT bleeding and ileus. Liver involvement typically manifests as cholestasis due to damage to the bile canaliculi, with elevated alkaline phosphatase and serum bilirubin. AGVHD is staged according to the extent of involvement of the skin, GIT, and liver. Severe AGVHD (grade III–IV) includes lower GIT AGVHD stage 2–4, liver involvement AGVHD stage 2–4, and stage 4 skin AGVHD (10). Severe GVHD is associated with a poor prognosis with a 5% long-term survival for grade 4 and 25% for grade 3 (11).

Corticosteroids are the first-line treatment for AGVHD, exhibiting potent immunosuppressive and anti-inflammatory effects. Despite this, a complete response is only witnessed in 25%–50% of patients with AGVHD. Both AGVHD and immunosuppressive medications increase the risk of infections (12) and other complications (13). Patients whose disease is refractory to corticosteroids have an unfavorable prognosis with increased TRM (14). There is no established consensus regarding standard second-line therapy for patients with steroid-resistant or steroid-dependent AGVHD, and options include intensification of systemic immunosuppressive therapy with different medications such as mycophenolate mofetil (MMF), anti-tumor necrosis factor (TNF) antibody therapy, or sirolimus (targeting the mammalian target of rapamycin inhibitors) (15).

Survival after HSCT has improved over recent years due to significant improvements in HLA typing techniques, less toxic conditioning regimens, and better supportive care and has led to 90% survival and cure in some nonmalignant diseases (11).

In this report, we describe the incidence, risk factors, and outcome of severe AGVHD in pediatric patients with nonmalignant diseases, following allogeneic HSCT in one single center over an 11-year period.

## 2. Materials and methods

### 2.1. Study population

We present a retrospective study, analyzing the outcomes of pediatric patients with nonmalignant diseases who underwent allogeneic HSCT from 2008 to 2019 and developed severe AGVHD (grade 3–4). The study was approved by the Institutional Review Board of Hadassah Medical Center.

### 2.2. Data collection

Data were collected from medical records and entered directly into password-protected Microsoft Excel spreadsheets (Redmond, WA, United States). The collected data included gender, age at HSCT, diagnosis, type and age of donor, source of stem cells, type of conditioning, engraftment details, stage and grade of AGVHD, treatment details, response to treatment, days of hospitalizations, and outcome. The primary goal of this study was to define incidence and outcome of severe AGVHD in pediatric patients with nonmalignant diseases following allogeneic HSCTs, as well as underscoring the multiple factors involved in the prognosis. In addition, we compared the survival rates of pediatric patients with nonmalignant diseases with severe AGVHD (grade 3–4), mild/moderate AGVHD (grade 1–2), and patients without AGVHD.

### 2.3. Definitions

We classified nonmalignant diseases into four groups: inborn errors of immunity, bone marrow failure, and other hematological and metabolic disorders. Donor types were divided into three groups based on HLA compatibility: matched (10/10), mismatched (8–9/10), and haploidentical (related donors <8/10); and based on relationship: sibling donor (SibD), family donor (FD), and UD. The HLA analysis was based on high-resolution genotyping.

Conditioning regimens were divided into three groups: reduced intensity (RIC), reduced toxicity, and myeloablative conditioning. The use of serotherapy in conditioning regimens was classified as: none, anti-thymocyte globulin (ATG), or alemtuzumab.

### 2.4. GVHD prophylaxis and treatment

Patients received GVHD prophylaxis with a combination of cyclosporine A (CsA) and MMF, the standard protocol for nonmalignant diseases in our center.

Acute and chronic GVHD were graded according to the modified Glucksberg criteria (16), and the revised Seattle criteria (17), respectively. In complicated and nonclassical cases, transjugular liver biopsy, or gastro/colonoscopy with biopsy was done to confirm the diagnosis of GVHD and exclude other reasons.

The first-line treatment in all patients was prednisolone at a dose of 2 mg/kg/day. Patients who responded to this dose of prednisolone after 5 days were defined as responsive, and patients who needed any escalation of steroid dose or addition of immunosuppressive second- and third-line treatments for AGVHD were defined as no responders.

### 2.5. Supportive treatment

All patients received trimethoprim/sulfamethoxazole and antifungal prophylaxis during and after transplantation. Patients with positive antibodies to herpes virus received prophylaxis with acyclovir starting from conditioning and continued until immunosuppressive therapy was stopped. Patients were isolated in rooms equipped with high-efficiency particulate air filter (HEPA) filters and received a regular diet. Additional supportive measures, such as total parenteral nutrition, blood component transfusions, and patient-controlled analgesia (PCA) pumps were administered when necessary. Polymerase chain reactions (PCRs) for cytomegalovirus (CMV), Epstein–Barr virus (EBV), and adenovirus were performed weekly, and in case of reactivations, preemptive antiviral treatment was started.

### 2.6. Statistical analysis

Analysis was done using the IBM SPSS Statistics, version 25.0 (Armonk, NY, United States). Descriptive statistics are given as the mean and standard deviation [M (SD)] or as a frequency with percentage [*n* (%)], according to the type of variable. Associations between different categorical factors were assessed by *χ*^2^ test and by Fisher's exact test. Comparisons between quantitative variables were assessed by *T*-test and nonparametric Mann–Whitney *U* test. All significant variables were entered into the multivariable logistic regression model. A two-tailed *p*-value <0.05 was considered statistically significant, *α* = 0.2. Survival analysis was done using the Kaplan–Meier estimator algorithm.

## 3. Results

### 3.1. Patients and transplant characteristics

A total of 247 pediatric patients with different nonmalignant diseases underwent 266 allogeneic bone marrow transplantations at Hadassah University Hospital between 2008 and 2019. Seventeen patients needed a second transplant and three patients required a third transplant.

Detailed patient and transplant characteristics of the entire cohort are presented in Table 1.

**Table 1:** Patient and transplant characteristics.

	Patients with grade 3–4 GVHD	Patients with grade 1–2 GVHD	Patients without GVHD	*p*-value
Number of patients	35	37	175	
Number of HSCTs	39	37	191	
Mean patient age, years	5.67	5.48	4.46	0.201
Median age, years (range)	5.00	3.42	2.99	
	(0.28–17.70)	(0.5–17.20)	(0.13–17.86)	
Mean donor age, years	28.21	24.27	18.82	<0.001
Median age, years (range)	28.00 (0.00–68.00)	21.88 (4.0–50.00)	16.50 (0.0–67.00)	
Gender: male/female	23/12	26/11	102/73	0.332
Diagnosis				
1. Inborn errors of immunity	7 (20%)	13 (35.1%)	73 (41.7%)	0.271
2. Bone marrow failure	9 (25.7%)	9 (24.3%)	38 (21.7%)	
3. Other hematological	4 (11.4%)	2 (5.4%)	17 (9.7%)	
4. Metabolic	15 (42.9%)	13 (35.1%)	47 (26.9%)	
Conditioning regimen				
1. Myeloablative	12 (34.3%)	17 (45.9%)	72 (41.1%)	0.198
2. Reduced intensity	3 (8.6%)	6 (16.2%)	37 (21.1%)	
3. Reduced toxicity	20 (57.1%)	14 (37.8%)	66 (37.7%)	
Type of donors				
1. Sibling donor	7 (20.0%)	14 (37.8%)	90 (51.4%)	<0.001
2. Family donor	6 (17.1%)	5 (13.5%)	51 (29.2%)	
3. Unrelated donor	22 (62.9%)	18 (48.6%)	47 (26.9%)	
Matched	20 (57.1%)	28 (75.7%)	142 (81.1%)	
Mismatched	15 (42.9%)	9 (24.3%)	21 (12.0%)	<0.001
Haploidentical	0	0	12 (6.9%)	
Source of stem cells				
1. BM	18 (51.4%)	28 (75.7%)	135 (77.1%)	0.023
2. PB	15 (42.9%)	9 (24.3%)	32 (18.3%)	
3. Cord	2 (5.7%)	0 (0%)	8 (4.6%)	
Use of lymphodepletion therapy				
1. No	1 (2.9%)	3 (8.1%)	14 (8.0%)	0.181
2. ATG	33 (94.3%)	27 (73.0%)	140 (80.5%)	
3. Campath	1 (2.9%)	7 (18.9%)	20 (11.5%)	
Chronic GVHD	13 (37.1%)	5 (13.5%)	4 (2.28%)	<0.001
Survival: yes/no	25/10	34/3	146/29	0.067
OS	71.4%	91.9%	83.4%	

GVHD, graft-vs.-host disease; HSCT, hematopoietic stem cell transplant; BM, bone marrow; PB, peripheral blood; ATG, anti-thymocyte globulin; OS, overall survival.

Of the 247 patients, 72 (29.1%) developed symptoms of AGVHD. Of those, 37 (14.9%) developed grade 1–2 (mild) AGVHD and 35 (14.1%) developed grade 3–4 (severe) AGVHD.

### 3.2. Risk factors for severe AGVHD (grade 3–4)

Thirty-five patients with AGVHD grade 3–4 underwent 39 transplantations, with four children undergoing two stem cell transplantations. All four patients who had undergone a repeat HSCT developed severe GVHD after the second HSCT. Median age was 5.0 years (range: 0.28–17.7), and 23 of the 35 (65.7%) patients were male.

Unrelated donor transplant was associated with an increased risk of severe AGVHD [62.9% in the severe AGVHD group vs. 48.6% and 26.9% in the mild and no AGVHD groups, respectively (*p* < 0.001)]. Mismatched donors were more commonly used in the severe AGVHD group [42.9% vs. 24.3% and 12% in the mild and no GVHD groups, respectively (*p* < 0.001)]. There was a statistically significant difference between age of the donor between groups; in patients with severe AGVHD, the median age was 28 years compared with 21.88 years in patients with mild AGVHD and 16.5 years in patients with no AGVHD (*p* < 0.001). Another risk factor for developing severe AGVHD was the use of PBSC as a stem cell source [42.9% in the severe AGVHD group vs. 24.3% and 18.3% in the mild and no AGVHD groups, respectively (*p* < 0.001)] (Table 1). When comparing the severe vs. no/mild AGVHD groups, the same risk factors were consistently apparent (Table 2). Other factors such as underlying diagnosis, type of conditioning regimen, use of immunosuppressive therapy, and age of the patient were not statistically significant.

**Table 2:** Risk factors for severe AGVHD vs. mild/no AGVHD.

	Patients with grade 3–4 GVHD	Patients without grade 3–4 GVHD	*p*-value
Type of donors			
Related donors (sibling + family)	13 (37.1%)	147 (69.3%)	<0.001
Unrelated donors	22 (62.9%)	65 (30.7%)	
Matched donor	20 (57.1%)	170 (85.0%)	<0.001
Mismatched donor	15 (42.9%)	30 (15.0%)	
Source of stem cells			
BM	18 (54.5%)	163 (79.9%)	0.002
PB	15 (45.5%)	41 (20.1%)	
Mean donor age, years	28.21	24.27	0.001^a^
Median age, years (range)	28.00 (0.00–68.00)	21.88 (4.0–50.00)	

GVHD, graft-vs.-host disease; BM, bone marrow; PB, peripheral blood.

^a^ Independent-samples Kruskal–Wallis Test.

### 3.3. Type of GVHD

The most common type of severe AGVHD in our cohort was GIT, with 9 patients developing isolated GIT AGVHD, 17 patients combined skin and GIT AGVHD, and 2 patients combined liver and GIT. Six children had multisystem AGVHD with GIT, skin, and liver involvement (Table 3). In most cases, skin and liver AGVHD appeared early after transplantation (within the first month) and responded well to steroid treatment. By comparison, GIT AGVHD developed beyond the first month post-transplant when symptoms of skin AGVHD resolved or improved. In most cases of GIT GVHD (23/34, 67.6%), patients had a poor response to steroids, prolonged course of treatment, and worse prognosis.

**Table 3:** Outcome of pediatric patients with nonmalignant diseases with GVHD stage 3–4.

Patients with GVHD stage 3–4	Survivors (*n* = 25)	Nonsurvivors (*n* = 10)	Impact on survival
Median age	5.71	4.98	*p* = 0.627
Gender male/female	16/9	7/3	*p* = 0.731
Diagnosis			
1. Immunodeficiency	6	1	* *
2. Bone marrow failure	5	4	*p *= 0.409
3. Hematological	4	2	* *
4. Metabolic or other rare genetic diseases	12	3	* *
Type of conditioning regimen			
1. Reduced intensity	2	1	*p = *0.492
2. Myeloablative reduced toxicity	14	6	* *
3. Myeloablative	9	3	* *
Type of donors			
1. Sibling donor	6	1	*p = *0.601
2. Family donor	3	3	* *
3. Unrelated donor	16	6	* *
Type of donors			
1. Matched	14	6	*p = *0.847
2. Mismatched	11	4	* *
Source of stem cells			
1. Bone marrow (+Cord blood)	17	3	*p = *0.016
2. Peripheral collection	8	7	* *
Age of donor years median (range)	29 (range 11–52)	33 (9–68)	*p = *0.445
Type of GVHD			
1. GIT	7	2	*p < *0.157
2. LIVER	1	0	* *
3. GIT + SKIN	13	4	* *
4. GIT + LIVER	2	0	* *
5. GIT + LIVER + SKIN	2	4	* *
Response to steroids			
1. Good steroid response	12	0	*p = *0.007
2. Bad steroid response (partial or nonresponse)	13	10	* *
Days of hospitalizations in 1st year(mean):	88 d	110 d	*p =* 0.425

GVHD, graft-vs.-host disease; GIT, gastrointestinal tract.

The most common therapy used for steroid nonresponders was pulse steroids (10 mg/kg/dose, 3–5 days), extracorporeal photophoresis (ECP), anti-TNF therapy, mesenchymal cells, methotrexate, Jak-1 inhibition, sirolimus, and Campath. Patients who responded well to first-line steroid treatment had significantly lower duration of admission during the first year following HSCT [48 days (range 30–72) compared to 159 days (range 61–280)] in the steroid-refractory group. It is interesting to note that all 13 patients with bad steroid response in the survivors group responded well to second- or third-line immunosuppressive therapy in contrast to 10 patients in the nonsurvivor group, who were refractory for all lines of GVHD treatment. The incidence of chronic GVHD was statistically significant higher in the group of patients with severe AGVHD, 37.1% vs. 13.5% and 2.28% in children with mild AGVHD and no AGVHD, respectively (*p* < 0.001) (Table 1).

### 3.4. OS and TRM of patients with severe AGVHD

**Figure 1:**
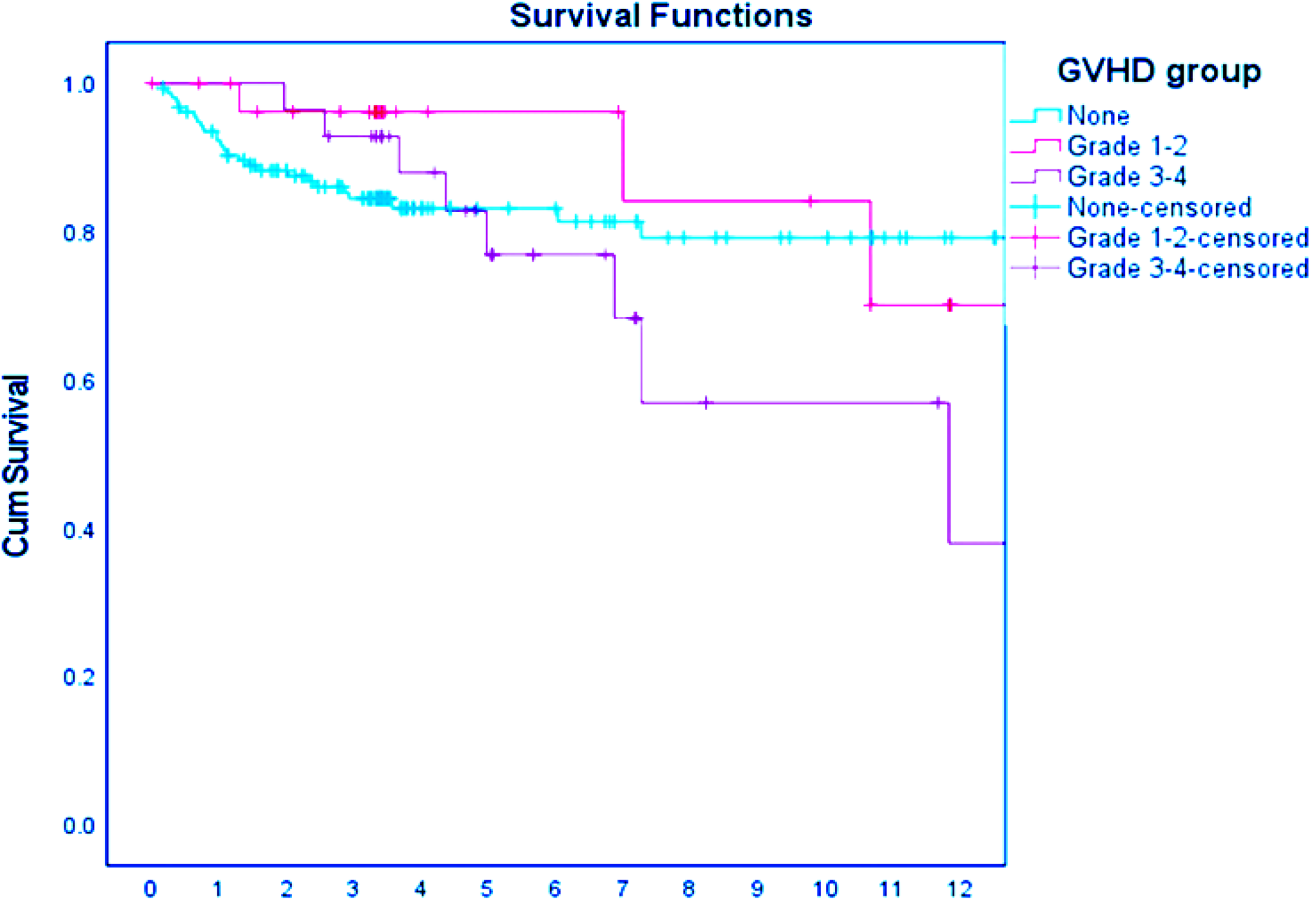
Survival according to AGVHD stage vs. no AGVHD.

**Figure 2:**
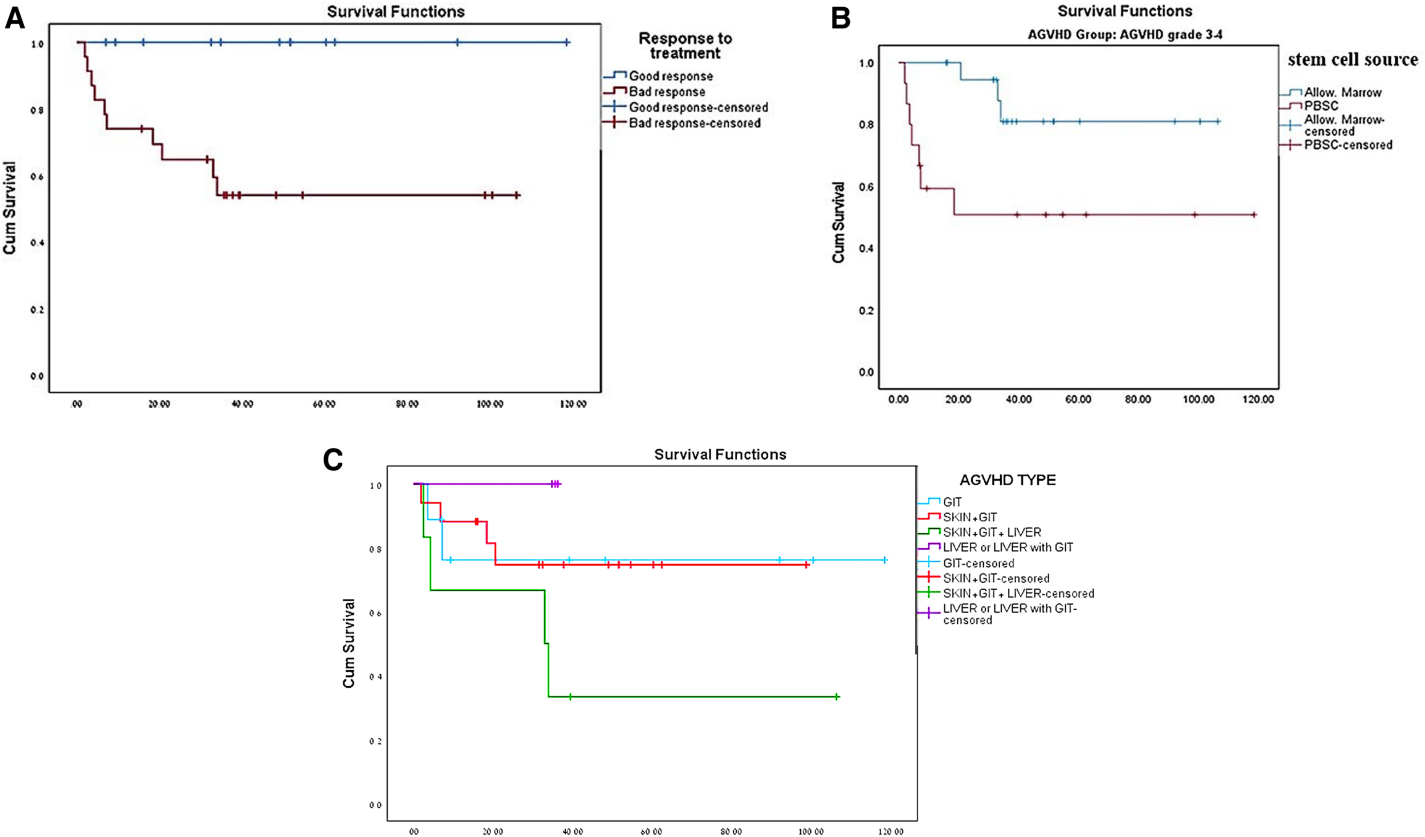
(**A**) Survival of patients with severe AGVHD based on prednisone response treatment. (**B**) Survival of patients with severe AGVHD based on stem cell source. (**C**) Survival of patients with severe AGVHD based on type of AGVHD.

Out of the 35 pediatric patients with severe AGVHD, 25 (71.4%) survived. The survival rate of pediatric patients with nonmalignant diseases with mild (grade 1–2) AGVHD was 91.9% and 83.4% for those without AGVHD, although this did not reach statistical significance (*p* = 0.067) (Table 1 and Figure 1). Ten of the 35 (28.6%) children in our cohort died from severe GVHD and complications of immunosuppressive treatment. All 10 nonsurvivors of severe AGVHD had GIT involvement, poor response to standard doses of steroids, and did not respond to second- or third-line therapy for GVHD (Tables 3, 4). Six patients were transplanted from UD, four from mismatched donors, and three after second transplantation (Table 4). Significant risk factors for mortality in pediatric patients with nonmalignant diseases and severe AGVHD were response to steroid treatment (*p = *0.007) (Figure 2A) and source of donor cells PBSC (*p = *0.016) (Figure 2B). The outcome of patients with severe AGVHD involving three systems was worse compared with other types of AGVHD, without reaching statistical significance (*p = *0.179) (Figure 2C). Other factors such as underlying diagnosis, type of conditioning regimen, type and matching of donor, and age of the patient and donor were not statistically significant (Table 3).

**Table 4:** Detailed characteristics of nonsurvivors.

*N*	Age (years)	Diagnosis	Year of BMT	*N* of BMT	Donor match, sex of donor, age of donor	Source of stem cells	Protocol	GVHD type response to steroids treatment	Time from BMT to death (months)	Reason for death
1	6.3	SAA	2012	2	8/10 MUD, Male, 22 years	PBSC	Campath	AGVHD stage 3 (Skin: good response GIT: poor response)	8	Sepsis, MOF
2	2.8	Hurler	2012	2	9/10 MUD, Female, 56 years	PBSC	Flu/Treo/TT/ATG	AGVHD stage 3 GIT: poor response	4	GVHD
3	9	Thalassemia	2012	1	9/10 MFD, Female, 33 years	BM	Flu/Treo/TT/ATG	AGVHD stage 4 Skin/GIT/liver: poor response	3	AGVHD, Pulmonary hemorrhage, MOF
4	5	Thalassemia	2015	1	10/10 MFD, Male, 68 years	PBSC	Flu/Treo/TT/ATG	AGVHD stage 4 Skin/GIT/liver: poor response Ext. CGVHD	4	Severe GVHD
5	2.28	MLD	2018	1	10/10 MUD, Female, 20 years	PBSC	Bu/Flu/ATG	AGVHD stage 4 Skin: good response GIT: poor response	18	Severe GVHD, progression of disease
6	8.6	SAA	2018	1	9/10 MUD, Female, 32 years	BM	Flu/Cy/TBI/ATG	AGVHD stage 4 Skin: good response GIT: poor response	19	Pneumococcal sepsis, MOF
7	1.87	HLH (Gricelli)	2018	1	10/10 MFD, Male, 30 years	BM	Flu/Treo-TT Campath	AGVHD stage4 Skin/Liver: good response GIT: poor response	34	Pneumococcal sepsis, MOF
8	2.26	ADA-2	2018	2	10/10 MSD, Male, 9 years	PBSC	Treo/CY-TBI/Campath	AGVHD stage 4 Skin/GIT: poor response	2	Sepsis, MOF AGVHD
9	10.4	Osteopetrosis	2019	1	10/10 MUD, Male, 33 years	PBSC	Flu/Treo/TT/ATG	AGVHD stage 4 GIT: poor response	7.5	Severe AGVHD, GIT bleeding, MOF
10	2.55	ADA-2	2018	1	10/10 MUD, Female, 24 years	BM	FLU/BU-TT/ATG	AGVHD stage 4 Skin/GIT/liver: poor response	57	Severe CGVHD, BO Sepsis

BMT, bone marrow transplantation; GVHD, graft-vs.-host disease; AGVHD, acute graft-vs.-host disease; CGVHD, chronic graft-vs.-host disease; SAA, severe aplastic anemia; MUD, matched unrelated donor; MMUD, mismatched unrelated donor; PBSC, peripheral blood stem cells; GIT, gastrointestinal tract; MOF, multiorgan failure; TT, thiotepa; TBI, total body irradiation; ATG, anti-thymoglobulin; MFD, matched family donor; BM, bone marrow; MLD, metachromatic leukodystrophy; HLH, hemophagocytic lymphohistiocytosis; ADA-2, adenosine deaminase 2 deficiency; BU, busulfan; BO, bronchiolitis obliterans.

## 4. Discussion

Allogeneic hematopoietic stem cell transplantation is often used to treat an increasing number of rare nonmalignant disorders. Despite significant advances in improved HLA typing, conditioning toxicity, and supportive treatment, AGVHD continues to be a major obstacle. There is no benefit of GVHD in patients with nonmalignant disorders, and any degree of GVHD in this population is considered an undesirable iatrogenic effect (3).

The incidence of AGVHD in pediatric patients with nonmalignant disorders varies between 15% and 30% and severe AGVHD (grade 3–4) about 9%–15% (3, 18).

In a large cohort of 183 pediatric patients with nonmalignant diseases from five centers in Florida, Horn et al. describe an incidence of AGVHD grade II–IV and AGVHD grade III–IV of 21.9% and 9.3%, respectively (3). Similar findings were reported from the Japanese transplant registry, which included all children with nonmalignant diseases transplanted between 1985 and 2016, with a cumulative incidence of AGVHD grade II–IV and AGVHD grade III–IV as 24% and 9.1%, respectively (18). Mahmoud et al. reported a 15% rate of AGVHD II–IV in a cohort of patients with nonmalignant hematological disorders undergoing HSCT (19). In our cohort, 29.1% of pediatric patients with nonmalignant diseases developed symptoms of AGVHD, and 14.9% had symptoms of AGVHD grade 1–2. The rate of severe AGVHD in our cohort was slightly higher than the aforementioned studies (14.1%), most probably due to a high incidence of patients receiving unrelated and mismatched grafts, and possibly our smaller cohort.

Severe GVHD continues to be a major source of morbidity and mortality following allogenic hematopoietic stem cell transplantation. Despite the availability of new immunosuppressive and antimicrobial drugs, the prognosis of grade 3–4 AGVHD continues to be dismal. In a cohort of patients with grade 3–4 AGVHD, 76% were steroid-refractory (20). A further review of the literature reveals steroid refractoriness in 100% of patients with grade III/IV AGVHD, requiring additional lines of treatment (20). Survival rates in the first year following HSCT were 0%–43% (21–23). In another large cohort of pediatric patients with AGVHD following HSCT for nonmalignant conditions, grade III/IV AGVHD was associated with poor 3-year OS (52.9% vs. 90.1% and 98.1% in patients without AGVHD and grade I/II AGVHD, respectively) (3).

Uygun et al. retrospectively analyzed 28 pediatric patients with stage III/IV GIT AGVHD. They observed skin AGVHD to be a typical initial manifestation AGVHD in the first 3 weeks following transplantation, while GIT AGVHD predominated after the second week following HSCT (1). Skin AGVHD demonstrated the best response to steroids. In this study, reported adult data showed survival of only 25%, and in children, the outcome was more favorable by about 55% (1). In our cohort, the most common type of severe AGVHD was GIT alone or in combination with other types. In accordance with previous studies (1), skin GVHD appeared early post-transplantation and was steroid-responsive in all cases, while most patients developed GIT AGVHD beyond the first month after engraftment when skin AGVHD improved or resolved and on immunosuppressive therapy. Most of these patients (67.6%) were steroid-refractory, needed multiple combinations of immunosuppressive therapy, had prolonged course of treatment, and had prolonged hospitalization.

Survival of patients with grade 3–4 AGVHD appears to have improved little in the last few years. El-Jawahri et al. performed a retrospective analysis of 427 patients with grade III–IV AGVHD and compared TRM and OS between years 1997–2006 and 2007–2012. They showed that 1-year TRM of patients with severe AGVHD declined from 58% in 1997–2006 to 38% in 2007–2012. As a result, 1-year OS increased from 30% in the early cohort to 42% in the most recent cohort (23). The reasons behind these improvements in the outcomes of patients with severe AGVHD are likely multifactorial and include better and more precise HLA typing for matched unrelated donor (MUD): improved prevention and treatment of infection complications and improved supportive care practice.

In our cohort, survival rate was 71.4% in patients with severe and refractory AGVHD. These rates are higher than previously described (3, 20–22) and are not significantly different from survival rates of patients with AGVHD stage 1–2 or no AGVHD. We attribute our improved survival rates to earlier initiation of additional immunosuppressive medications in steroid-refractory cases, combined treatment with the use of immunosuppressive drugs with other methods of treatment as ECP and mesenchymal cells, close monitoring for infections, and intensive supportive treatment with preventive antifungal and antiviral medications. The most significant impact to mortality in our cohort of pediatric patient with nonmalignant diseases and severe AGVHD were source of stem cells and poor response to steroid treatment.

We recognize the study's limitations, mainly its retrospective nature, and involving only single center. The main strength of our study is this relatively large group of pediatric patients with nonmalignant diseases over an 11-year period and the evaluation of risk factors for severe AGVHD and prognosis.

In conclusion, this retrospective analysis of AGVHD in pediatric patients transplanted for nonmalignant conditions recapitulates the significance of mismatched unrelated donor (MMUD) and PBSC as risk factors for severe AGVHD, and steroid responsiveness as a prognostic marker in these patients. It also demonstrates a favorable outcome compared with the previously published literature.

## Data Availability

The raw data supporting the conclusions of this article will be made available by the authors, without undue reservation.
